# Res-RBG Facial Expression Recognition in Image Sequences Based on Dual Neural Networks

**DOI:** 10.3390/s25123829

**Published:** 2025-06-19

**Authors:** Xiangwei Mou, Yongfu Song, Xiuping Xie, Mingxuan You, Rijun Wang

**Affiliations:** 1 College of Electronic and Information Engineering/Integrated Circuits, Guangxi Normal University, Guilin 541004, China; yfsong@stu.gxnu.edu.cn (Y.S.);; 2 Teachers College for Vocational and Technical Education, Guangxi Normal University, Guilin 541004, China

**Keywords:** facial expression recognition, feature extraction, deep learning, Bi-GRU, residual structure, attention mechanism

## Abstract

Facial expressions involve dynamic changes, and facial expression recognition based on static images struggles to capture the temporal information inherent in these dynamic changes. The resultant degradation in real-world performance critically impedes the integration of facial expression recognition systems into intelligent sensing applications. Therefore, this paper proposes a facial expression recognition method for image sequences based on the fusion of dual neural networks (ResNet and residual bidirectional GRU—Res-RBG). The model proposed in this paper achieves recognition accuracies of 98.10% and 88.64% on the CK+ and Oulu-CASIA datasets, respectively. Moreover, the model has a parameter size of only 64.20 M. Compared to existing methods for image sequence-based facial expression recognition, the approach presented in this paper demonstrates certain advantages, indicating strong potential for future edge sensor deployment.

## 1. Introduction

Facial expression recognition research can be traced back to the 1970s. American psychologist Ekman [1] categorized human facial expressions into six types: anger, surprise, fear, happiness, sadness, and disgust. Currently, most facial expression recognition research is focused on the extraction of features and the classification of these six types of expressions. The research process of facial expression recognition based on neural networks can be divided into two stages: facial expression recognition based on static images and facial expression recognition based on image sequences.

In facial expression recognition algorithms based on static images [2,3], the algorithms extract facial expression features from individual images, offering advantages such as lower computational complexity and straightforward feature extraction. Over the past few decades, these algorithms have seen widespread development. However, static expression recognition overlooks the dynamic nature of expressions. Feature extraction in static approaches focuses solely on spatial features, neglecting the extraction of temporal features, and it exhibits certain differences from facial expressions in real-world scenarios and lacks strong robustness. From this perspective, researching facial expression recognition based on image sequences holds significant importance for the application and development of facial expression recognition technology. Facial expression recognition algorithms based on image sequences [4,5] utilize continuous sequential images as the dataset, taking into account feature information in both spatial and temporal dimensions. In real-world scenarios, this method has advantages over static classification methods.

Therefore, scholars have also conducted research in the field of facial expression recognition using image sequences. Liu et al. [6] proposed a method called Main Direction Mean Optical (MDMO) flow, which primarily normalizes and statistically analyzes features of the region of interest on the face. This method takes into account the local motion and spatial position information of micro-expressions, demonstrating a certain level of robustness to facial translation, head pose variations, and lighting changes in facial expression sequences. Suk et al. [7] employed active shape models (ASM) to track and model facial feature points. They used the displacement between neutral facial feature points and non-neutral feature points as dynamic features. A support vector machine (SVM) was then employed for facial expression classification. This approach achieved good accuracy in real-time expression detection. Zhao et al. [8], building upon local binary patterns (LBPs), proposed the Local Binary Pattern on Three Orthogonal Planes (LBP-TOP) to investigate dynamic facial expression recognition. This method effectively extracts LBP features at three different levels, constructing a global description of facial expression motion. Although the aforementioned traditional methods have low computational and training sample requirements and can effectively extract facial expression features, these methods have poor generalization and cannot extract deep features, and the feature extraction methods are manually designed, consuming time and effort. In recent years, convolutional neural networks (CNNs) have demonstrated strong generalization and feature extraction capabilities in static image facial expression recognition research. To enable convolutional neural networks to continue playing a role in facial expression recognition research involving image sequences, Tran et al. [9] improved the convolutional neural network by introducing the three-dimensional convolutional neural network (C3D). This modification enhances the network’s capability to process features with a temporal dimension. Compared to two-dimensional convolutional neural networks, this network exhibits greater flexibility in extracting temporal features. Based on this approach, Liu et al. [10] proposed a C3D with deformable motion constraints, allowing for the recognition and effective encoding of specific facial movements within this structured network with deformable constraints. Zhang Ruijun et al. [11] introduced residual skip neural modules and attention mechanisms into a three-dimensional convolutional neural network, improving the accuracy of facial expression recognition based on image sequences. Jiang Wan et al. [12] designed a dual-path model that combined a C3D with a long short-term memory (LSTM) network. Both pathways of the network simultaneously extracted spatial and temporal information, enabling the learning of finer facial expression features. However, despite the ability of three-dimensional convolutional neural networks to handle facial expression recognition in image sequences effectively, they suffer from issues such as large network parameter sizes and high computational complexity. Therefore, Khorrami et al. [13], based on the powerful image feature extraction capabilities of CNNs and the good temporal information memory capabilities of RNNs, developed cascaded CNN and RNN networks, which achieved the recognition of dynamic facial expressions. However, traditional RNN may not effectively learn the relevant information between the preceding and succeeding frames in image sequences, leading to lower recognition rates. Therefore, Cheng Huanxin et al. [14] replaced the RNN with an improved RNN, resulting in the LSTM network [15]. This approach effectively extracts temporal features between frames in image sequences, achieving better facial expression classification results. However, aiming to enhance the facial expression recognition efficiency, the further increase in the network depth and LSTM unit quantity has led to the problem of vanishing gradients. This issue has hindered improvements in the facial expression recognition efficiency based on image sequences, impacting the further application and development of facial expression recognition technology.

In response to the aforementioned issues, this paper proposes a facial expression recognition method for image sequences based on the fusion of dual neural networks (Res-RBG). The main contributions are as follows:

(1) To fully extract facial expression features in both spatial and temporal dimensions and achieve facial expression recognition in image sequences with few network parameters and low computational complexity, a hybrid network is proposed by cascading the ResNet50 and GRU networks;
(2) To fully utilize temporal information between sequences, promote the integrity of the information flow throughout the network, and address issues such as gradient vanishing, this paper introduces a bidirectional GRU network based on the GRU network; additionally, following the principles of residual structures, an RBG network is designed;
(3) To effectively enhance the network’s ability to extract key features and improve the efficiency of facial expression recognition in image sequences, this paper embeds an improved lightweight and efficient channel attention module (G-ECANet) into the ResNet50 network.

## 2. Related Work

### 2.1. The Model of Facial Expression Recognition

Based on the discussion of other methods for facial expression recognition based on image sequences, and considering both the accuracy and speed of recognition, this paper selects the CNN for its strong capabilities in image feature extraction and the RNN for its ability to capture temporal information. This combination allows for the consideration of both spatial and temporal features in facial expression recognition from image sequences. To maintain a balance between accuracy and efficiency, lighter versions of the CNN and RNN are chosen as the basic networks for this research. Furthermore, improvements are made to these basic network models to enhance the efficiency of the recognition process. Thus, based on these considerations, the specific choice for the basic research model in this paper is as follows.

#### 2.1.1. Spatial Feature Extraction Network

The task of visual object recognition has increasingly utilized CNNs, such as AlexNet [16], VGG [17], GoogleNet [18], ResNet [19], and others. In the pursuit of improved recognition accuracy, many approaches rely on increasing the depth of neural networks to achieve higher accuracy. However, as the depth of the convolutional layers increases, a common issue arises during network training: the vanishing gradient problem. This problem makes it difficult for shallow layers to update the parameters, thereby hindering the updating of the parameters in these layers. Therefore, Kaiming He et al. [20] proposed the residual neural network (ResNet) in 2015. Compared to regular CNNs, ResNet utilizes a special network structure known as shortcut connections. It incorporates the concept of residual learning by directly passing input information to the output, preserving the integrity of the information and simplifying the learning process. It addresses, to a certain extent, the issues of information loss and degradation in traditional CNNs, and it also helps to mitigate problems such as vanishing gradients and network degradation in deep networks. Therefore, considering factors such as model feature extraction capabilities and network parameters, this paper adopts the ResNet50 network as the basic network for spatial feature extraction.

#### 2.1.2. Temporal Feature Extraction Network

LSTM networks, with their extended memory capacities, have consistently outperformed traditional RNN models across various sequential tasks [21]. Importantly, LSTM is less prone to the vanishing gradient problem. However, LSTM structures are relatively complex, resulting in higher computational costs and larger model parameter sizes. Research has found that the forget gate is the most crucial gating mechanism in LSTM. In fact, simplified networks containing only the forget gate have been shown to outperform standard LSTM networks on multiple benchmark datasets. Among the array of simplified LSTM variations, the GRU [22] is one of the most widely used variants of RNNs. It has a smaller computational cost and shorter training time. The GRU combines the internal state vector and the output vector into a unified state vector. It also reduces the number of gates to two: the reset gate and the update gate. It has a smaller computational load and shorter training time. Moreover, the model’s recognition efficiency is not poor, and there is ample room for improvement in the network. Therefore, this paper selects the GRU network as the basic network for the extraction of temporal features of facial expressions.

### 2.2. Dataset and Data Preprocessing

#### 2.2.1. Dataset

**Figure 1:**
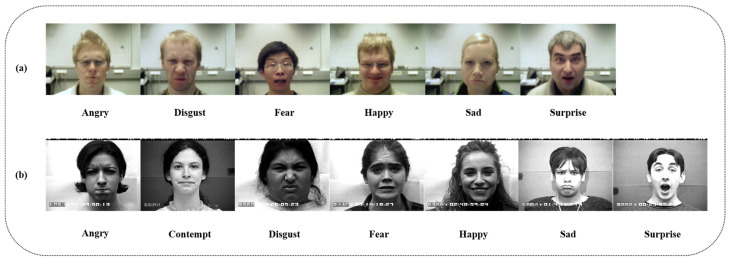
Partial sample images from image sequence facial expression dataset: (**a**) CK+ dataset, (**b**) Oulu-CASIA dataset.

To ensure the reproducibility of the experimental results, this paper uses the average data of ten model training sessions as the foundation for the evaluation of the model. Figure 1 shows some sample images from the two facial expression datasets used in this paper: (a) represents partial samples from the CK+ facial expression dataset, and (b) represents partial samples from the Oulu-CASIA facial expression dataset.

CK+ [23]: The Extended Cohn-Kanade (CK+) dataset is the most representative dataset in facial expression recognition, consisting of 593 video sequences from 123 participants and 327 video sequences from 118 participants, all marked according to 7 basic expressions: anger, contempt, disgust, fear, happiness, sadness, and surprise. The length of the sequence varies between 10 and 60 frames. Each video sequence starts with a neutral expression and ends with a peak expression.

Oulu-CASIA [24]: The Oulu-CASIA dataset contains 2880 image sequences, obtained under three lighting conditions—strong, normal, and weak light—as well as two imaging systems: near-infrared and visible light. Among them, 480 sequences were obtained under visible light conditions and were all labeled with 6 basic expressions, namely anger, disgust, fear, happiness, sadness, and surprise. Similar to CK+, each sequence in this dataset starts with a neutral expression and ends with a peak expression.

#### 2.2.2. Data Preprocessing

Considering that the input dimensions of neural networks are generally fixed, this paper normalizes the lengths of two facial expression image sequences to a predetermined value. Taking into account the average sequence length of the two sequences and the requirements of network training, the image sequences in this study are normalized to a length of 16 frames. If the number of frames in an image sequence is greater than 16, the frames are uniformly sampled at intervals to obtain 16 frames. If the number of frames is less than 16, the last frame of the sequence is duplicated to complete the 16 frames. This approach is employed because, in the two datasets used in this study, the facial expression changes in each image sequence follow a process from a neutral state to a peak expression, and the frames capturing the peak expression are more indicative of the overall emotional change. Moreover, the MTCNN [25] facial detection algorithm is utilized to detect the facial region in the image sequences and to locate the positions of facial key landmarks. By rotating the facial images to ensure that both eyes are at the same horizontal level, facial alignment is achieved. Finally, the detected facial images are cropped to a size of 224 × 224 to minimize irrelevant factors around the face (such as hair, jewelry, and facial contours) that may affect facial expression recognition, thereby enhancing the accuracy of facial expression recognition.

**Figure 2:**
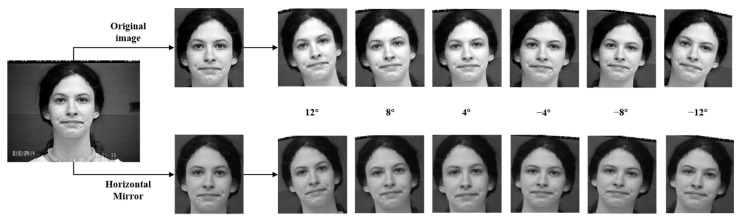
Facial cropping and data augmentation process.

**Figure 3:**
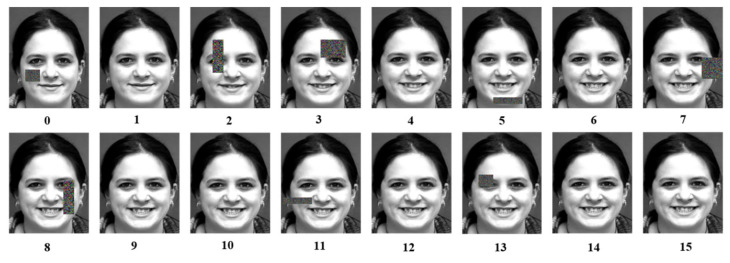
Random masking of facial expression sequences.

Deep neural networks require an adequate number of training samples to ensure strong generalization; however, currently, there is a scarcity of samples in image sequence facial expression datasets, leading to overfitting issues during training. Therefore, this paper employs data augmentation techniques to augment the number of facial expression image sequences, enhancing the model’s generalization ability and mitigating overfitting. First, the image sequences are horizontally flipped. Then, the original dataset and the horizontally flipped dataset are rotated at angles of −12°, −8°, −4°, 4°, 8°, and 12°. In the end, the number of data samples is increased by 14 times compared to the original dataset. The process of facial cropping and data augmentation is illustrated in Figure 2. To further enhance the model’s generalization, this paper employs the method of random masking on the augmented facial expression image sequences [26], as shown in Figure 3. The distribution of sequence samples across different categories before and after data preprocessing is shown in Table 1.

## 3. Models and Methods

**Figure 4:**
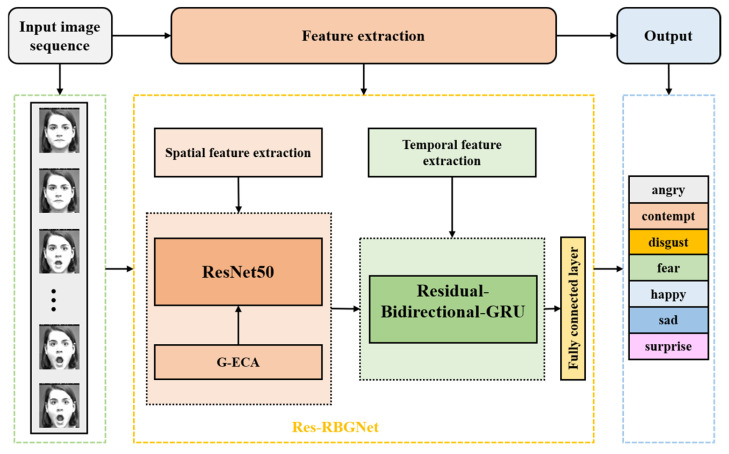
The overall architecture of the Res-RBG network.

To achieve image sequence facial expression recognition, this paper proposes a method for image sequence facial expression recognition based on the fusion of dual neural networks (Res-RBG). Firstly, to fully extract facial expression features in both spatial and temporal dimensions, and to achieve facial expression recognition in image sequences while maintaining a relatively low network parameter count and computational load, this paper proposes a method that combines the ResNet50 and GRU networks. Secondly, to make full use of temporal information between sequences, promote the integrity of the information flow within the network, and prevent issues such as gradient disappearance, this paper replaces the original GRU network with a Bi-GRU network. Furthermore, based on the principle of residual structures, an RBG network is designed in this paper. Finally, to effectively enhance the network’s ability to extract key features and improve the efficiency of facial expression recognition in image sequences, the improved lightweight and efficient channel attention module (G-ECANet) is embedded into the ResNet50 network in this paper. The overall architecture of the constructed Res-RBG network is shown in Figure 4.

### 3.1. The Design of the G-ECANet Network

**Figure 5:**
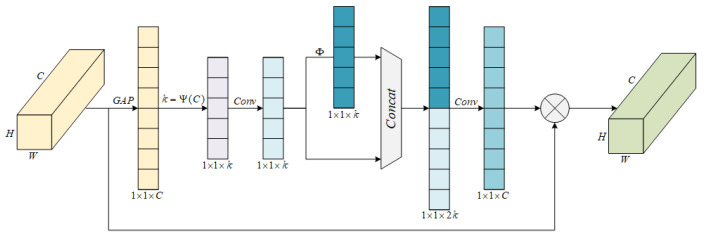
The network structure of G-ECANet.

The introduction of channel attention mechanisms in convolutional neural networks contributes to improvements in model performance [27]. For example, the squeeze-and-excitation network (SENet) can adaptively learn global information and assign different weight proportions to each channel based on the importance of channel feature information, thereby improving the efficiency of model feature extraction [28]. ECA [29] is a lightweight and efficient channel attention module, an optimized version of SENet. It mainly optimizes the SENet module from two perspectives—dimension reduction and cross-channel information interaction—overcoming the contradiction between model performance and complexity [30] and effectively improving the model’s performance. To efficiently obtain richer spatial feature information and improve the efficiency of extracting subsequent temporal information, the improved ECA attention module (G-ECANet) based on the Ghost idea [31] is introduced into the ResNet50 network in this paper. The specific module structure is shown in Figure 5.

The Ghost idea involves splitting the traditional convolution into two steps. Using fewer computations, it generates a smaller-channel feature map through traditional convolution. Then, through linear transformation with fewer computations, it generates a new feature map. Finally, the two sets of feature maps are concatenated. The specific design is as follows.

Firstly, the input feature map with dimensions H×W×C (H represents the height of the feature map, W represents the width, and C represents the number of channels) undergoes global average pooling, compressing it into a 1×1×C feature map. This feature map is then convolved to obtain a feature map of size 1×1×k. The size of the convolution kernel used in the convolution is adaptively determined through a non-linear mapping on the channel dimension. The adaptation function is defined as follows:

(1) $$k=\psi (C)={\left|\frac{\log_{2}(C)}{\gamma }+\frac{b}{\gamma }\right|}_{odd}$$

In the formula, k represents the size of the convolution kernel (taken as an odd number), C is the number of channels, and b and γ are used to change the ratio between the number of channels C and the size of the convolution kernel. In layers with a large number of channels, a larger convolution kernel is used to enhance the extraction of input channel feature information. In layers with a small number of channels, a smaller convolution kernel is used to reduce cross-channel interaction and thereby reduce the computational complexity.

Using the Ghost idea, the first step involves performing a regular convolution operation with a convolution kernel of size k to obtain the intrinsic feature map $Y\in R^{1\times 1\times k}$. The computational formula is as follows:

(2) $$Y=X*C_{k}+v$$

In the equation, ∗ represents the convolution operation, v is the bias term, and $C_{k}$ represents the convolution kernel of size k, reducing the output channel number to further compress the original features.

The second step involves using the generated intrinsic feature map Y for a simple linear transformation Φ, resulting in the Ghost feature map. The linear transformation formula is as follows:

(3) $$Y_{i}^{\prime }=\Phi (Y_{i}),\;(i=1,\;\ldots ,\;n)$$

In the formula, $Y_{i}$ is the i-th intrinsic feature map in Y, Φ is the linear operation performed on the intrinsic feature map, and $Y_{i}^{\prime }$ represents the Ghost feature map obtained after the i-th intrinsic feature map undergoes the transformation.

Finally, the two sets of feature maps are concatenated through a concatenation operation [32], resulting in a feature map with size 1×1×2k. Then, through the convolution of 1×1, channel feature learning is performed, resulting in a feature map of 1×1×C channel attention. This feature map is then subjected to element-wise multiplication with the feature map of the original input H×W×C, and the channel attention feature map with the same size as the original input feature map is output. The proposed G-ECANet in this paper adopts the Ghost idea, using linear transformation instead of convolutional operation for the efficient fusion of channel features while avoiding dimension reduction when learning channel attention information. This effectively enhances the efficiency of extracting spatial feature information in the model.

### 3.2. The Design of the RBG Network

**Figure 6:**
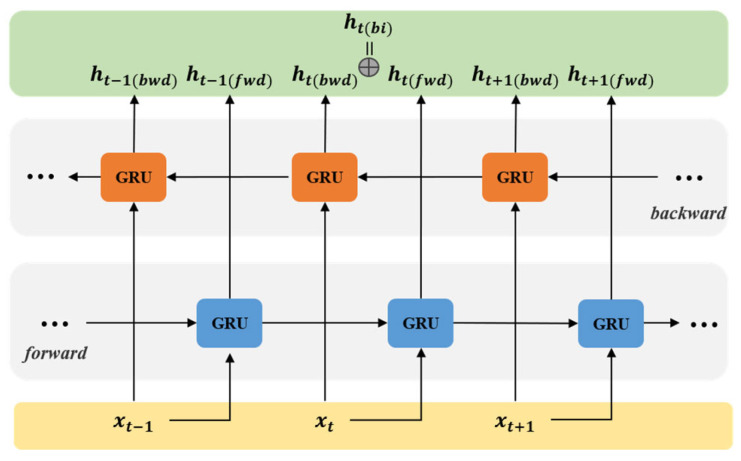
The network structure of Bi-GRU.

The traditional GRU network can only rely on the unidirectional propagation of information when learning facial expression temporal features. It fails to fully utilize information from both the past and future directions, leading to the insufficient extraction of temporal features and impacting the efficiency of image sequence facial expression recognition. Therefore, this paper employs a bidirectional propagation Bi-GRU network, where each time step contains information from both the past and the future. This enables the network to fully leverage the temporal correlation between frames in the image sequence, learning more effective features. The structure of the Bi-GRU network is shown in Figure 6, consisting of two unidirectional GRUs with opposite directions. The forward GRU moves from the beginning to the end of the sequence, while the backward GRU moves from the end to the beginning of the sequence. The computational formula for their outputs is as follows:

(4) $$h_{t(fwd)}=G_{fwd}(x_{t},h_{t-1(fwd)})$$

(5) $$h_{t(rwd)}=G_{rwd}(x_{t},h_{t+1(rwd)})$$

(6) $$h_{t(bi)}=h_{t(fwd)}⊕h_{t(rwd)}$$

where $h_{t(fwd)}$ represents the state of the forward GRU, $h_{t(rwd)}$ is the state of the backward GRU, and the symbol ⊕ denotes the concatenation of the two states $h_{t(fwd)}$ and $h_{t(rwd)}$, resulting in the output $h_{t(bi)}$ of the Bi-GRU.

**Figure 7:**
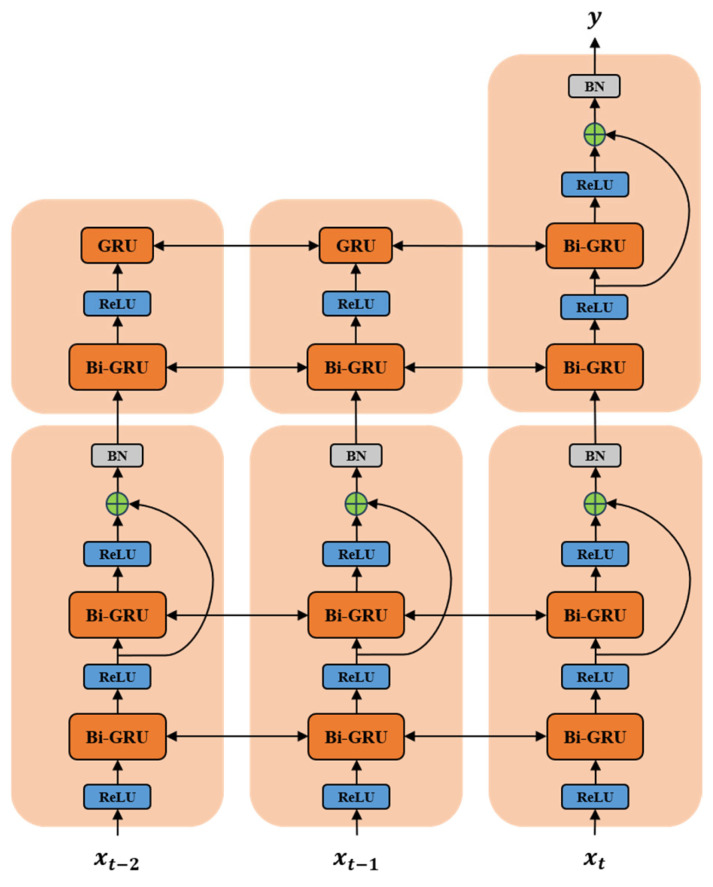
The network structure of RBG.

Although the Bi-GRU network can improve the accuracy of facial expression recognition to some extent, the issue of gradient vanishing becomes more pronounced with an increase in the number of GRU network units. Therefore, based on the excellent idea of residual networks, this paper proposes an RBG network, which integrates the residual concept into the Bi-GRU network. This enhances the ability to learn temporal features in the Bi-GRU network while avoiding the issue of gradient vanishing that arises with too many GRU network units. This further improves the accuracy of facial expression recognition in the model. The structure of the RBG network is shown in Figure 7, and the pseudocode of the RBG model algorithm is shown in Algorithm 1. As depicted in the figure, information flows both horizontally (temporal dimension) and vertically (spatial dimension). Apart from the input and output layers, the network comprises two residual blocks serving as hidden layers. Each residual block consists of 2 Bi-GRU units, resulting in a total of 8 GRU units. This paper adopts the ReLU activation function. The RBG network can integrate features across adjacent frames, capture accumulated temporal information over the entire sequence of facial expressions, and ultimately use a softmax classification layer to determine the category of the final expression.
**Algorithm 1** RBG Neural Network**Input**: X ∈ ℝ^(T×d_in)    // Input sequence of length T with d_in featuresn_layers = 6       // Total number of stacked layersd_hidden        // Hidden state dimensiontraining_flag     // Boolean for train/inference mode**Output**: y ∈ ℝ^(T×d_out)    // Output sequence1: // Initialization2: **for** l ← 1 to n_layers **do**3:    **if** l ≤ 2 **then**4:      W_gru[l] ← Initialize GRU Weights(d_hidden)5:    **else**6:      W_bn[l] ← Initialize BatchNorm Params()7:      W_gru[l] ← Initialize Bi-GRU Weights(d_hidden)8:    **end if**9: **end for**10: // Forward pass11: h ← X12: **for** l ← 1 to n_layers **do**13:    **if** l > 2 **then**14:      h ← BatchNorm(h, W_bn[l], training_flag)15:    **end if**
16:    h ← ReLU(h)  // Element-wise activation17:    **if** l ≤ 2 **then**18:      h ← GRU(h, W_gru[l])  // Unidirectional GRU19:    **else**20:      h ← BiGRU(h, W_gru[l])  // Bidirectional GRU21:    **end if**22: **end for**23: y ← LinearProjection(h)  // Final output layer24: return y

### 3.3. The Construction of the Dual Neural Network Res-RBG

**Figure 8:**
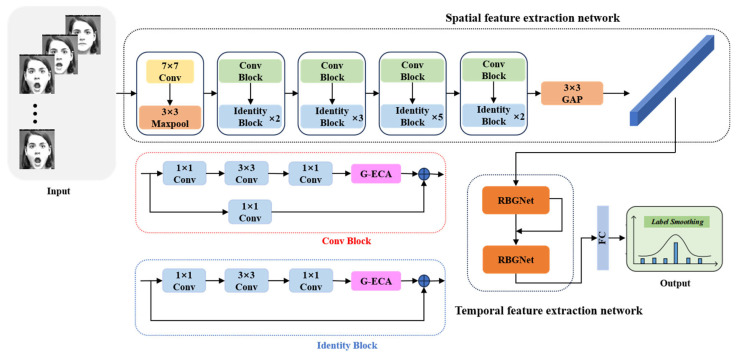
The network structure of Res-RBG.

To enhance the efficiency of facial expression recognition in image sequences in real-world scenarios, this paper, based on the previously described network modules, constructs a model for image sequence facial expression recognition using a dual neural network (Res-RBG) fusion approach. The model structure of the Res-RBG network is shown in Figure 8. In this paper, the ResNet50 network and the GRU network are used as the base networks for spatial and temporal feature extraction, respectively. In terms of spatial feature extraction, to efficiently obtain richer spatial feature information and enhance the network’s ability to extract key features, the improved G-ECANet attention module is introduced into the ResNet50 network in this paper. In terms of temporal feature extraction, this paper replaces the unidirectional GRU network with a bidirectional Bi-GRU network to fully utilize information from both the past and future directions, promoting the integrity of the information flow in the network. In addition, on the basis of the Bi-GRU network, a residual bidirectional GRU (RBG) network is proposed, integrating the residual idea to address the issue of gradient vanishing that may arise with an excessive number of GRU network units, thus enhancing the quality of temporal feature extraction in the network. The proposed model in this paper fully extracts spatial features from the facial expression image sequence. The extracted spatial features are sequentially input into the RBG network to comprehensively extract temporal features from the facial expression image sequence. Finally, the softmax classifier outputs the recognition result. The model achieves the efficient recognition of facial expressions in image sequences with relatively few network parameters and low computational complexity.

## 4. Experiments and Analysis

In order to validate the accuracy and effectiveness of the proposed image sequence facial expression recognition model based on dual neural networks, the CK+ and Oulu-CASIA datasets—two image sequence facial expression datasets—are used for parameter selection, ablation, and comparative experiments in this paper. All experiments are conducted using the PyTorch 1.12.1 deep learning framework, trained and tested on Pycharm. The hardware configuration includes the Win10 operating system, an Intel Core i5-12490F (2.9 GHz CPU, 32 GB RAM), and an NVIDIA GeForce RTX 3080 (10 GB) graphics card.

### 4.1. Experimental Evaluation Indicators

In multi-class classification problems, when the true value of the data is positive and the model’s predicted value is also positive, it is referred to as true positive (TP). Similarly, there are false positive (FP), true negative (TN), and false negative (FN). Common evaluation metrics in facial expression recognition datasets include accuracy and the confusion matrix. Accuracy represents the proportion of correctly predicted samples to the total number of samples, as shown in the formula below. The confusion matrix provides a detailed representation of the classification accuracy for each expression category.

(7) $$Accuracy=\frac{TP+TN}{TP+TN+FP+FN}$$

In order to better evaluate the recognition performance of the Res-RBG model, this section introduces the $F_{1}$ score as a new evaluation metric. The $F_{1}$ score, which considers both precision and recall, is commonly used in the evaluation of multi-class problems and provides a balanced assessment, reflecting the model’s robustness. The specific formula is as follows:

(8) $$precision=\frac{TP}{TP+FP}$$

(9) $$recall=\frac{TP}{TP+FN}$$

(10) $$F_{1}=2\cdot \frac{precision\cdot recall}{precision+recall}$$

### 4.2. Network Parameter Settings

This paper adopts the ResNet50 network pretrained on ImageNet [33] as the initialization parameters for the model. It then fine-tunes it on the relevant dataset, and a fully connected layer with a softmax classifier is trained. The experiments utilize the Adam optimizer for network optimization, with the batch size set to 16. The weight decay is configured as 1 × 10^−4^, and the learning rate is set to 0.0001. An exponential decay method is employed, reducing the learning rate by a factor of 0.9 every 10 epochs and training 300 epochs on two datasets, respectively. In the Res-RBG network, the choice of the number of layers in the RBG network and the number of neurons in the GRU hidden layer in the RBG network can have a significant impact on the model’s recognition results. Therefore, under certain conditions regarding other parameter settings, this paper, considering practical model training configurations, discusses the facial expression recognition accuracy on the CK+ and Oulu-CASIA datasets for different numbers of layers in the RBG network and various numbers of neurons in the GRU hidden layer.

#### 4.2.1. Determination of RBG Network Layers

In the case of a sufficient number of data samples, the choice of the number of layers in the RBG network influences the model’s recognition efficiency. In theory, a larger number of layers tends to improve the model’s recognition performance; however, it also increases the number of network parameters and the computational complexity. Considering the limitations of the hardware configuration for model training, this paper conducts comparative experiments with one-layer, two-layer, and three-layer RBG networks on the CK+ and Oulu-CASIA datasets. The experimental results are presented in Table 2.

The table reveals that, compared to the one-layer RGB network, the recognition accuracy of the two-layer RGB network is increased by 0.8% and 0.74% on the CK+ and Oulu-CASIA datasets, respectively. Similarly, the three-layer RGB network improves the recognition accuracy by 1.04% and 0.79% on the CK+ and Oulu-CASIA datasets, respectively. However, the training time required for stable recognition accuracy in the two-layer and three-layer RGB networks is 1.09 times and 1.23 times that of the one-layer RGB network, respectively. Taking into account the cost and efficiency of network training, this paper selects the Res-RBG model that integrates the two-layer RGB network.

#### 4.2.2. Determination of the Number of Neurons in the Hidden Layer of GRU

**Figure 9:**
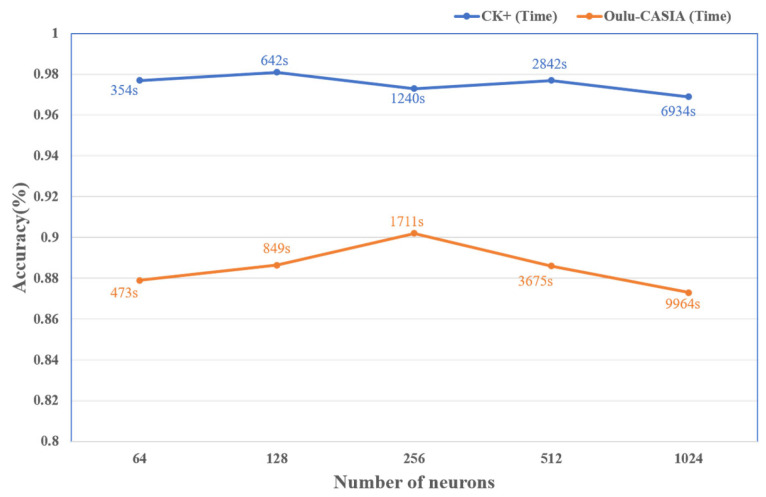
Network performance under different numbers of neurons.

In this paper, the spatial image features extracted by ResNet50 are sequentially aggregated and then fed into a two-layer RBG network, consisting of layers with 64, 128, 256, 512, and 1024 neurons, to extract temporal features from the image sequences. For different numbers of neurons, experiments are conducted under the condition of keeping other parameters constant on both the CK+ and Oulu-CASIA datasets. The experimental results are presented in Figure 9.

From the above graph, it can be observed that, when the number of GRU neurons is set to 128, the model achieves better recognition accuracy on both the CK+ and Oulu-CASIA datasets. This suggests that the information output by the current RBG network can better characterize the temporal dynamics of the facial expressions, making it more discriminative for the recognition task. Additionally, when the recognition accuracy stabilizes, the training time for 64 and 128 neurons is relatively lower. Considering both the recognition accuracy and training time, the network is most efficient when the number of neurons is set to 128. Therefore, this paper selects 128 neurons for the GRU hidden layer.

### 4.3. Ablation Experiments

In order to analyze the contributions of each network module proposed in this paper to the overall model, this paper uses the ResNet50-GRU network as the baseline model. Random masking (RM) represents the model with random masking applied to the dataset. Baseline+Bi-GRU represents the model with Bi-GRU replacing the unidirectional GRU. Baseline+RBG represents the use of a fused residual structure, namely the Bi-GRU network, in the model to replace the original Bi-GRU network. Baseline+RBG+ECA represents the model with the ECA module integrated into the spatial feature extraction network, i.e., ResNet50. Baseline+RBG+G-ECA represents the model with the improved G-ECA module replacing the ECA module. This paper conducts ablation experiments on both the CK+ and Oulu-CASIA datasets, comparing the recognition accuracy and $F_{1}$ score as performance metrics. The experimental results are shown in Table 3.

**Figure 10:**
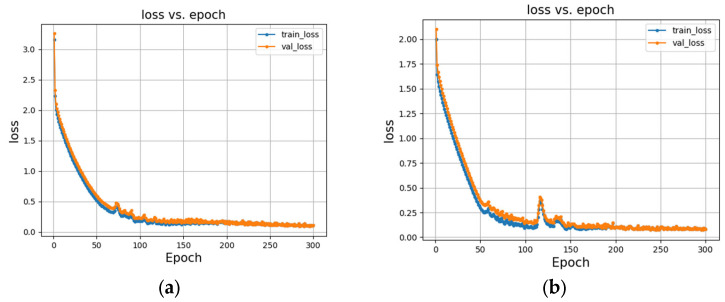
Loss curves: (**a**) the loss curve on the CK+ dataset; (**b**) the loss curve on the Oulu-CASIA dataset.

In Table 3, it can be seen that, after RM processing, the recognition accuracy of the baseline on the CK+ and Oulu-CASIA datasets increases by 0.38% and 0.29%, respectively. Compared to the baseline model, the introduction of Bi-GRU improves the recognition accuracy on the CK+ and Oulu-CASIA datasets by 0.46% and 0.55%, respectively. The addition of the fused residual structure in the RBG network does not show a significant improvement in the recognition accuracy but results in a notable increase in the $F_{1}$ score, indicating the enhanced robustness of the model. The integration of the ECA module brings a slight improvement in both the overall recognition accuracy and $F_{1}$ score. Finally, the incorporation of the improved G-ECA module leads to further improvements in the recognition accuracy and $F_{1}$ score. In comparison to the baseline model, our model’s recognition accuracy is enhanced by 1.20% and 2.32% on the CK+ and Oulu-CASIA datasets, respectively. Through the ablation experiments, the efficacy of the proposed approach in recognizing facial expressions within image sequences is validated. The training loss curve of the proposed model is shown in Figure 10, demonstrating successful convergence on both datasets.

**Figure 11:**
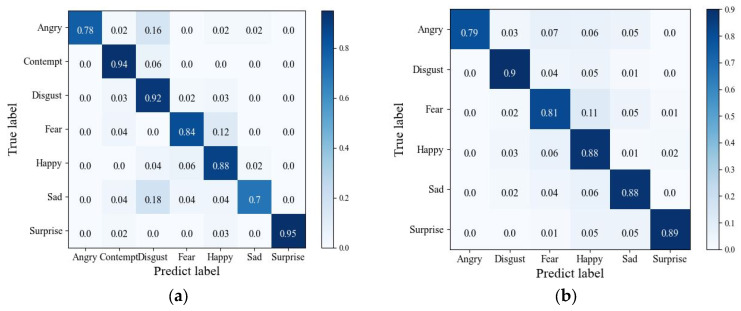
Confusion matrix: (**a**) confusion matrix on the CK+ dataset; (**b**) confusion matrix on the Oulu-CASIA dataset.

To more accurately investigate the recognition capabilities of the proposed method for various facial expressions in image sequences, we conduct ten-fold cross-validation on our model and then generate confusion matrix plots for the CK+ and Oulu-CASIA datasets, as shown in Figure 11. For the CK+ dataset, our model achieves the highest recognition accuracy for expressions of contempt and surprise, while the accuracy for other expressions follows closely behind. Despite the similarities in facial features such as eyebrows and mouth corners among the expressions of anger, sad, and disgust, our proposed method strengthens the information channels for key features and enhances the dependencies between key temporal features. This allows the model to effectively classify expressions that may be easily confused or have less distinct features. For the Oulu-CASIA dataset, the overall recognition accuracy is lower compared to the CK+ dataset due to the presence of more real-world disturbances. Among the expressions, disgust achieves the highest recognition rate, followed by surprise, sadness, and happiness, with accuracy rates of 89%, 88%, and 88%, respectively. The model still demonstrates strong stability in recognizing these expressions despite the challenges posed by the dataset’s environmental factors. Overall, the method proposed in this paper exhibits good discriminative capabilities for facial expressions in image sequences.

### 4.4. Comparative Experiments

To further validate the proposed method’s efficiency in recognizing facial expressions within image sequences, comparative experiments are conducted on the CK+ and Oulu-CASIA datasets, comparing the proposed method with state-of-the-art methods in recent years. The differences in recognition accuracy between the proposed method and other methods are presented in Table 4.

In Table 4, for the CK+ dataset, it can be observed that the proposed method achieves accuracy of 98.10% for facial expression recognition within image sequences. Compared to methods such as STM-ExpLet, HCNN-LSTM, DTAGN, LOMo, IDT+FV, FN2EN, MELADA, and CLIP, the proposed method shows an improvement in recognition accuracy of approximately 1.3–13.23%. Compared to advanced methods like DeRL, Inception-w, and WMCNN-LSTM, the proposed method also demonstrates a slight advantage in recognition accuracy. Compared to PPDN, PHRNN-MSCNN, ARDfee, ESTLNet, DCNN, STANER, and ORAC-Net, the proposed method exhibits slightly lower recognition accuracy. However, the GRU network used in this paper is more lightweight and flexible than networks like LSTM, resulting in fewer parameters and shorter training times for the proposed model, making it more practical for real-world applications. For the Oulu-CASIA dataset, the proposed method achieves recognition accuracy of 88.64%. Compared to methods such as Atlases and STM-ExpLet, the proposed method shows a significant improvement in recognition accuracy, which is approximately 14% higher. Compared to advanced methods like DTAGN, PPDN, LOMo, MELADA, and CLIP, the proposed method also demonstrates a certain degree of advantage. Methods like DeRL, MMN, ESTLNet, WMCNN-LSTM, STANER, and TSGCN exhibit good recognition accuracy, especially for the TSGCN method; its recognition accuracy reaches 91.30%, which is 2.66% higher than that of the method proposed in this paper. However, this method improves the model recognition accuracy by increasing the network depth, with a much larger number of model parameters than the 64.20M proposed in this paper and a more complex model structure with higher computational costs. In conclusion, in the field of facial expression recognition in image sequences, the proposed method in this paper has certain advantages compared to some other advanced methods, taking into account both the overall recognition accuracy and the difficulty of model training.

## 5. Conclusions

This paper introduces an image sequence facial expression recognition method based on the fusion of dual neural networks (Res-RBG). Building upon the ResNet50 network and the GRU network, facial expression features in image sequences are extracted from both spatial and temporal dimensions. Replacing the GRU network with the RBG network incorporating fused residual structures enhances the integrity of the information flow through the network, preventing issues such as gradient vanishing. Moreover, the ResNet50 network incorporates an improved lightweight and efficient channel attention module (G-ECANet), effectively enhancing the network’s ability to extract crucial feature information. The experimental results indicate that the proposed method achieves high recognition accuracy on both the CK+ and Oulu-CASIA datasets, demonstrating certain advantages compared to existing methods. However, the proposed method also has some limitations. For instance, its effectiveness is more pronounced on datasets like CK+ (experimental datasets under ideal conditions), while, on datasets like Oulu-CASIA (datasets in real-world scenarios), the model’s recognition efficiency is relatively lower due to the influence of various real-world disturbances. Therefore, future research should focus more on the study of facial expressions in image sequences in real-world scenarios to enhance the practical application value of facial expression research.

## Figures and Tables

**Table 1:** Sequence sample distribution (pre-/postprocessing).

Dataset/Number of Sequences		Anger	Contempt	Disgust	Fear	Happiness	Sadness	Surprise	Sum
CK+	Original Sequence	45	18	59	25	69	28	83	327
	Data Augmentation	630	252	826	350	966	392	1162	4578
	Random Masking	630	252	826	350	966	392	1162	4578
Oulu-CASIA	Original Sequence	80	-	80	80	80	80	80	480
	Data Augmentation	1120	-	1120	1120	1120	1120	1120	6720
	Random Masking	1120	-	1120	1120	1120	1120	1120	6720

**Table 2:** Results under different layers of RBG.

Model	CK+		Oulu-CASIA	
	Accuracy (%)	Time (s)	Accuracy (%)	Time (s)
ResNet50-1RBG	97.30	574.2	87.90	775.3
ResNet50-2RBG	98.10	641.5	88.64	848.8
ResNet50-3RBG	98.34	881.8	88.69	953.7

**Table 3:** Performance comparison of different modules of network.

Model	CK+		Oulu-CASIA	
	Accuracy (%)	$F_{1}$	Accuracy (%)	$F_{1}$
Baseline	96.90	0.576	86.32	0.639
Baseline + RM	97.28	0.539	86.61	0.616
Baseline + RM + Bi-GRU	97.36	0.564	86.87	0.629
Baseline + RM + RBG	97.39	0.713	86.91	0.681
Baseline + RM + RBG + ECA	97.77	0.734	87.76	0.693
Baseline + RM + RBG + G-ECA (ours)	98.10	0.744	88.64	0.704

**Table 4:** Performance comparison of different methods.

Model	CK+ (%)	Improvement	Oulu-CASIA (%)	Improvement
STM-ExpLet [34]	94.20	+3.90	74.59	+14.05
HCNN-LSTM [10]	84.87	+13.23	-	-
DTAGN [35]	97.30	+8.00	81.46	+7.18
PPDN [36]	99.30	−1.20	84.59	+4.05
LOMo [37]	95.10	+3.00	82.10	+6.54
IDT+FV [38]	95.80	+2.30	-	-
FN2EN [39]	96.80	+1.30	87.70	+0.94
PHRNN-MSCNN [40]	98.50	−0.40	86.25	+2.39
ARDfee [41]	98.70	−0.60	-	-
DeRL [2]	97.30	+0.80	88.00	+0.64
Inception-w [42]	97.80	+0.30	85.41	+3.23
ESTLNet [43]	99.04	−0.94	89.38	−0.74
WMCNN-LSTM [44]	97.50	+0.60	88.00	+0.64
SCAN [45]	97.31	+0.79	86.56	+2.08
DCNN [46]	98.46	−0.36	88.54	+0.10
STANER [47]	98.23	−0.13	89.52	−0.88
Atlases [48]	-	-	75.52	+13.12
MMN [49]	-	-	88.30	+0.34
ORAC-Net [50]	98.20	−0.10	89.70	−1.06
MELADA [51]	96.80	+1.30	82.40	+6.24
TSGCN [52]	-	-	91.30	−2.66
CLIP [53]	94.20	+3.90	86.90	+1.74
Res-RBG (ours)	98.10		88.64	

## Data Availability

The datasets utilized in this study, namely CK+ and Oulu-CASIA, can be accessed at the following respective URLs: CK+—“https://sites.pitt.edu/~emotion/ck-spread.htm” (accessed on 20 February 2024); Oulu-CASIA—“https://cstr.cn/16666.11.nbsdc.qmxrtsgv” (accessed on 25 February 2024).
